# Measuring Nursing Productivity Based on a Review of the Literature: A Pilot Study

**DOI:** 10.1002/hsr2.73218

**Published:** 2026-09-09

**Authors:** Elahe Farzan, Zhila Najafpour, Efat Jahanbani, Mohamad Arezomand

**Affiliations:** ^1^ Department of Health Care Management, School of Public Health Ahvaz Jundishapur University of Medical Sciences Ahvaz Iran; ^2^ Social Security Organization Ahvaz Khuzestan Province Iran

**Keywords:** hospital, measure, nurse, productivity, time motion study

## Abstract

**Background and Aims:**

One of the performance indicators of nurses is measuring their productivity. This is despite the fact that there is a serious weakness regarding the criteria to measure nursing productivity. Therefore, this study aimed to define the productivity indicators and to conduct a pilot test using the productivity measures in a hospital.

**Methods:**

This multi‐phase study is conducted in three parts. In the first phase, a review is conducted to extract nurses' productivity measures. In the second phase, the measures are prioritized and evaluated using experts' opinions. Finally, a pilot study based on the list of productivity measures is implemented. For this reason, we conducted a time‐motion study to analyze work efficiency in a pilot hospital.

**Results:**

In the systematic review phase, 11 articles were included in the study. They reported several measures like actual working hours, expected working hours, nurses' salaries and benefits, the number of patients under care, and relative value units (RVU) as indicators to measure nurses' productivity. Experts rated output/input, standard hours/actual hours, productive hours in patient day, and actual hours as more feasible, valid, and important indicators to measure productivity for nurses. Finally, in the third phase of this study, in order to measure actual working hours, nursing activities were extracted. Thirty‐six nurses were observed and evaluated in three internal, surgical, and special care units. The mean monthly clinical time was highest in the Internal Medicine unit (116.17 h), followed by the Surgical unit (109.93 h), and the Intensive Care unit (106.83 h). The ICU had the highest standard, recorded (Timex‐based), and paid hours. Finally, productivity was calculated by dividing clinical work hours by attendance hours in units. Performance ratio (productivity) was about 77%, 75%, and 68% in the internal medicine, surgical, and intensive care units, respectively.

**Conclusions:**

The productivity status of nurses was at a desirable level. The average productivity of nurses in the internal unit was higher than others. The productivity index can be the criterion of action in the field of performance‐based payment.

## Highlights

- Inadequate definitions and measures of nursing productivity are still controversial.
- Actual working hours, expected working hours, nurses' salaries and benefits, the number of patients under care, and relative value unites (RVU) are the indicators to measure nurses' productivity.
- Productivity was calculated by dividing clinical work hours by attendance hours in units.
- The productivity status of nurses was at a desirable level.

**Abbreviations**
FTE: full‐time equivalent
HRH: human resource for health
ICU: intensive care unit
RVU: relative value unit

## 1 Background

Over the past decades, the number of nurses has rapidly grown globally, with currently over 3.35 million full‐time equivalents (FTEs) in 2018 [1]. Consequently, as more nurses enter the health system, evidence demonstrates an increasing rate of quality and safety of nursing care [2], patient satisfaction [3], and access to health care [4]. Although nurses' role has evolved on providing quality care to patients, the need to demonstrate nurses' productivity becomes essential.

Productivity has been defined as a measure of output per unit of input. The input of human resources for health (HRH) may be established through measures of the workforce or salary, whereas their output assessment is complex. Productivity is only one aspect of healthcare systems; it has been suggested that productivity measures should be related to quality and health outcomes [5, 6, 7]. However, this may be challenging at the institutional level, where multiple treatment procedures and patient groups are pooled. Past studies confirmed that the link between hospital efficiency, quality of care, and productivity varies from positive associations to mixed results [8, 9]. Productive staff contributes to the hospital attaining its goals, reducing medical errors, leaving no care task undone, improving the effectiveness of care, and reducing turnover and absenteeism [10].

Within the nursing discipline, productivity should consider patients' needs, nursing competencies, the availability of material resources, and services. However, assessment of HRH productivity is complex because of the multiple tasks (i.e., patient treatment, teaching, and research), also differences among specialties regarding diversity in patient care levels. Studies considered nursing productivity based on different concepts like patients' acuity, rationing of nursing care, fair allocation of resources, and nursing workload [11, 12, 13]. Additionally, nursing productivity is usually quantified as the cost of required care for a defined number of patients. The literature considers nursing workload a direct reflection of the mentioned variables and affects the delivery of patient care, patient safety, and satisfaction of nurses and patients [14, 15].

However, inadequate definitions and measures of nursing productivity are still controversial. Based on our knowledge, there is a need for more clarity and consistency in how nursing productivity should be measured. For health care managers is essential to create new mechanisms, processes, and structures to measure productivity across the care continuum. Our research team aimed to extract nursing productivity measures and to assess the measures in a pilot study to calculate productivity levels.

## 2 Methods

This multi‐phase study was conducted in three parts: a literature review, an expert panel, and a pilot study. A list of productivity measures was initially extracted based on a review and then validated by an expert panel. After that, a survey based on the finalized list of productivity measures was developed in three different types of units (i.e., surgical, internal medicine, and intensive care units) in a pilot hospital.

### 2.1 Phase 1: Literature Review

In the first phase, a systematic review was conducted to respond to the first research question: How should nursing productivity be measured? The review was conducted in accordance with PRISMA guidelines. Electronic databases including Embase, PubMed, Scopus, ProQuest were searched from inception to 2021.

The search strategy combined terms related to nursing workforce and productivity, including health personnel, health manpower, health professional, health workforce, human resource, productivity, effectiveness, efficacy, and indicator (See Supporting Information S1: Tables S1–S4). Studies were eligible if they explicitly defined criteria or indicators used to measure nursing productivity. Studies that did not provide explicit productivity indicators or focused on non‐nursing personnel were excluded.

All retrieved records were imported into EndNote, and duplicates were removed. Titles and abstracts were independently screened by two reviewers (Z.N. and E.F.), followed by full‐text assessment for eligibility. Data were extracted using a researcher‐developed form, including author, year, country, study setting, study design, study period, and productivity measures with their definitions (See Supporting Information S1: Table S5).

A qualitative synthesis was conducted using content analysis to identify and categorize nursing productivity indicators based on conceptual similarity. The methodological quality of included studies was assessed using the Joanna Briggs Institute (JBI) critical appraisal tools (see Supporting Information S1: Table S6). Any disagreements were resolved through discussion.

### 2.2 Phase 2: Measures Validation

An initial list of nursing productivity measures was developed based on the findings of the literature review conducted in the previous phase. This list was subsequently subjected to content validation by an expert panel comprising seven individuals, including two head nurses, two nurse managers, and three academicians in nursing and health care management. Eligibility criteria included holding a professional role in nursing practice, nursing management, or nursing academia. Panel members were purposively selected based on predefined inclusion criteria, including: (1) a minimum of 5 years of professional experience in the related fields; (2) prior scholarly or practical experience related to nursing productivity or performance measurement; and (3) willingness to participate in all study rounds.

Prior to rating, the definitions of all proposed indicators were reviewed and refined to enhance clarity and shared understanding. Panel members then independently assessed each indicator using a five‐point Likert‐type scale ranging from 1 (strongly disagree) to 5 (strongly agree), evaluating feasibility (applicability), validity, and importance. Ratings were completed individually and collected anonymously to reduce potential group influence. Panel members were also invited to provide qualitative feedback and suggestions through open‐ended questions. A predefined consensus threshold of 70% agreement was applied for each indicator. Indicators that did not meet this threshold in the first round were re‐evaluated in a second round. Indicator prioritization was determined based on mean importance scores. Quantitative data were entered into Microsoft Excel and analyzed using descriptive statistics to summarize expert agreement. The resulting validated and prioritized list of productivity measures was then finalized for implementation in the subsequent phase of the study.

### 2.3 Phase 3: Pilot Study and Time–Motion Observation

A pilot study was conducted to evaluate the feasibility, clarity, and applicability of the finalized nursing productivity measures in a real clinical setting. The pilot was not designed to generate generalizable estimates; rather, its purpose was methodological testing. To calculate the time of nursing activities, a prospective and observational time and motion study was performed in three hospital units (surgical, internal medicine, and intensive care) within a public hospital. Thirty‐six nurses were randomly selected from eligible nursing staff across these units.

Nursing activities were identified through a combination of direct clinical observation and a literature review, resulting in a comprehensive activity taxonomy (See Supporting Information S1: supplementary 4). All activities performed by nurses during observed shifts were included, and individual nursing activities constituted the unit of observation. Observations were conducted across different units and scheduled across multiple shifts according to a predefined timetable to capture routine variation in workflow and workload patterns. Observation durations ranged from 7 to 12 h, corresponding to actual shift lengths.

After obtaining informed consent, two observers followed each nurse throughout their assigned shifts. Observations were conducted based on a predetermined schedule. Each unit was monitored for at least 2 weeks. Time spent on each activity was recorded using a stopwatch, with seconds as the base unit and subsequently converted to minutes. To address variability in observation duration across shifts, all time‐based measures were standardized and expressed as proportions of total observed working time per nurse per shift. When multiple nurses performed an activity simultaneously, the full duration of the activity was recorded and multiplied by the number of participating nurses. Data were collected over a 3‐month period in 2022, yielding a total of 120 observation hours on weekdays.

To ensure consistency between observers, inter‐rater reliability was assessed through simultaneous data collection. A third observer temporarily joined the primary observers to evaluate agreement. Reliability was defined as a percentage agreement with discrepancies of less than 5%. Additionally, a trained researcher monitored all nursing activities across different shifts for 1 day to validate the quality of the recorded data. In cases where certain activities occurred infrequently, interviews were conducted with nurses. To reduce potential Hawthorne effects inherent in direct observational studies, several mitigation strategies were applied. Observers remained present in the units for extended periods to allow nurses to acclimatize to their presence. Data collection was distributed across different nurses, units, and shifts to minimize systematic behavior change associated with repeated observation.

For data analysis, individual observed activities were first coded according to the predefined activity taxonomy and then aggregated into higher‐order activity categories. Aggregation was performed by summing time spent on individual tasks within each category and calculating the proportion of total working time allocated to each category per shift.

Nursing productivity calculation:

Productivity was calculated using the following approach.

- Nurses' working hours (based on Timex records): Total working hours were extracted from the Timex attendance recording system.
- Clinical working hours (based on a time–motion study): Clinical working hours were estimated based on the time spent on each nursing care activity, the frequency of care per patient, the average number of patients assigned to each nurse, and the number of monthly shifts per nurse.
- Paid working hours: Paid hours for nursing staff were calculated, including base salary, overtime, and performance‐based bonuses.
- Annual leave days: Annual leave was adjusted and converted into monthly hours for analysis.
- Non‐productive hours: Hours related to public holidays, leave, sick leave, training time, and meeting time were deducted from total working hours.

Required data were collected from various hospital units. Payroll information included payments for monthly worked hours (productive time) and payments for vacations, holidays, sick leave, maternity leave, and other paid absences (nonproductive hours) for nurses. Salary and benefits data were obtained from the hospital accounting department, while nurses' working hours were extracted from the hospital's human resources department. Descriptive statistics were used to calculate overall productivity levels.

Finally, nurses' productivity was calculated by dividing clinical working hours by physical presence hours, as well as by dividing clinical working hours by paid working hours. Based on these calculations, productivity scores were categorized as follows: less than 50% was considered low productivity, 50%–75% as moderate productivity, and greater than 75% as high productivity.

## 3 Results

### 3.1 Literature Review

**Figure 1:**
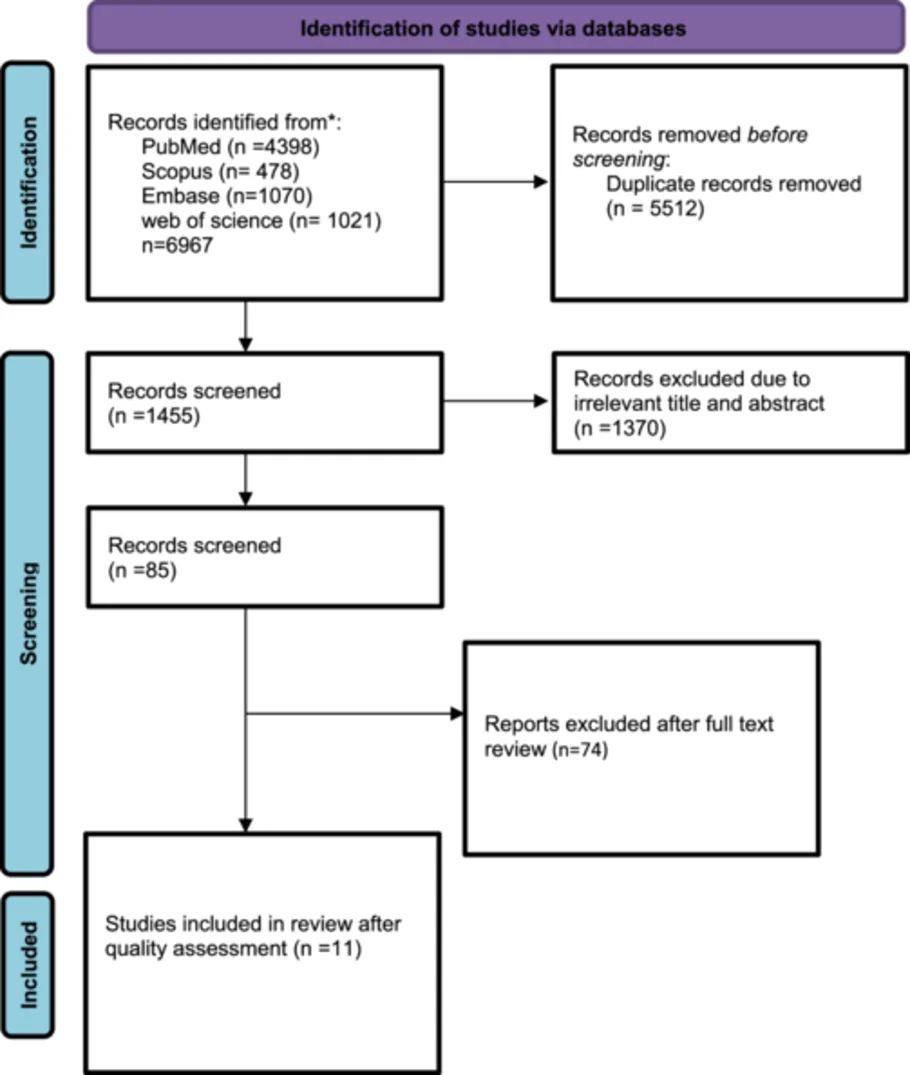
PRISMA flow chart.

Out of 1822 articles obtained from the systematic search, a total of 1455 studies were eligible for title and abstract screening after removing duplicates (344). Eighty‐five studies were selected for full‐text screening, and 11 articles were included in this review) [16, 17, 18, 19, 20, 21, 22, 23, 24, 25, 26]. Meanwhile, Table 1 shows the extracted productivity Indicators from the included articles (See Figure 1).

**Table 1:** Extracted productivity indicators.

Measures	Definition
Output/input	Output: patient care hours per patient day Input: salaries and benefits paid to nurses
Standard hours/actual hours	Standard hours: nursing workload (according to the percentage of actual time spent at the patient's bed) Actual hours: productive hours (sum of working hours − non‐productive hours) Non‐productive hours: holidays, vacations, sickness, training time, meeting time
Actual hours/expected hours	Output: working hours of patient care by nurse (time measurement) Required working hours (based on the nurse's salary)
Relative value units	The relative value of the services calculated based on the patient bill, the number of patients, and the relative value of the services provided per patient in the shift
Relative value units	The relative value of services calculated based on patient billing, number of patients
Relative value units	The relative value of nursing services over full‐time clinical equivalents
Productive hours*patient day	The number of hours with full interest (in a working day) in the number of sick days in a period of time (2 weeks) ☑ Calculate the number of unproductive hours ☑ Adjusted productivity calculation ☑ Calculation of overtime
Relative value units	The relative value of the services provided based on the patient's bill
Actual hours	Nurses' self‐declaration of the amount of time spent by the type of nursing services
Actual hours/expected hours	Actual hours: productive hours (sum of working hours − non‐productive hours) Non‐productive hours: holidays, vacations, illness, training time, meeting time

### 3.2 Measures Validation

In the second phase, the extracted measures were rated by expert panels. Based on their opinion, six productivity measures, including output/input, standard hours/actual hours, productive hours in patient day, and actual hours, were rated as more feasible, valid, and important measures and were considered to calculate nursing productivity in the third phase (see Tables 2 and 3).

**Table 2:** Evaluation of productivity measurement indicators from the perspective of feasibility, validity, and importance.

Productivity indicator	Feasibility	Validity	Importance	Mean
Output/input	4.71	4.86	4.71	4.76
Standard hours/actual hours	4.43	4.29	4.43	4.38
Actual hours/expected hours	3.43	4.71	4.57	4.24
Relative value units	2.0	3.10	3.10	2.73
Productive hours ∗ patient day	4.71	4.29	4.29	4.43
Actual hours	4.57	2.86	3.43	3.62

**Table 3:** Productivity measurement for prioritization.

	Percentage of priorities assigned to indicators					
Extracted indicator Productivity indicator	First priority	Second priority	Third priority	Fourth priority	Fifth priority	Sixth priority
Output/input	57%	28%	14%	—	—	—
Standard hours/actual hours	28%	57%	—	14%	—	—
Actual hours/expected hours	—	14.3%	42.9%	42.9%	—	—
Relative value units	—	—	—	—	28%	71%
Productive hours ∗ patient day	14.3%	14.3%	28.6%	28.6%	14.3%	0%
Actual hours	—	—	—	14.3%	57.1%	28.6%

### 3.3 To Calculate Productivity Measures

The sample consisted of 36 nurses who were women. The average age was 36.46 years old with an average of 11.5 years of working experience. All of them had a bachelor's degree in nursing sciences and worked full‐time.

Nursing activities were classified into four categories only: direct patient care, collective patient care, unit‐related tasks, and personal‐related tasks.

- Direct patient care is defined as care that can be directly related to one specific patient and involves communication between the nurse and the patient. This category includes 14 activity groups (i.e., handing out medication, wound care, communication with patients or their families, and so on). At the same time, indirect nursing care is the specific patient‐related services that do not require communication between the nurse and patient. This category includes documentation and communication with other units.
- Collective patient care is defined as patient‐related tasks that are impossible to attribute to a specific patient. This category includes three activity groups (i.e., general preparation of medication, specimen collection, and patient handover).
- Unit‐related tasks include five activity groups (i.e., education, meetings, administrative duties, domestic duties, checking mobile trolley, and incident reporting).
- Personal‐related tasks include three activity groups (such as breaks, walking time, and others) (See more details in nurses' activities in Supporting Information S1: Table S7).

In total, we observed 2540 nursing care measures measurements (944 in surgical, 1051 in internal medicine, 545 in ICUs). Based on the time–motion phase of the study, the average time allocated to nursing activities was calculated. It should be noted that not all activities are performed for every patient during a single shift. Therefore, the estimated times vary according to the frequency of activities and the number of patients assigned to each nurse across different units. To determine the clinical time, the frequency of nursing activities and the number of patients under each nurse's care were identified, and the final clinical time was calculated accordingly. The average clinical time spent per shift was 391.31 min for the Internal Medicine unit, 381.4 min for the Surgical unit, and 340.78 min for the Intensive Care Unit (See Table 4). The mean clinical time monthly was highest in the Internal Medicine unit (116.17 h), followed by the Surgical unit (109.93 h), and the Intensive Care unit (106.83 h).

**Table 4:** Average clinical time per shift (based on time‐motion study in minutes).

	Internal medicine	Surgical	ICU
Average clinical time spent per shift	391.31	381.4	340.78
Average clinical time spent during the morning shift	362.85	353.76	367.73
Average clinical time spent during the evening shift	283.96	314.89	277.37
Average clinical time spent during the night shift	527.14	475.55	377.24

Workload and time allocation varied across units. The ICU had the highest standard, recorded (Timex‐based), and paid hours, while the surgical unit generally showed the lowest values. Paid hours exceeded both standard and actual recorded hours in all units (Table 5).

**Table 5:** Monthly nursing workload and time allocation (average).

	Internal medicine	Surgical	ICU
Standard working hours	189	184.01	198.98
Working hours based on Timex records	150.27	144.66	155.52
Paid hours	210.33	207.77	216.43
Non‐productive hours	2.66	2.97	2.18
Number of shifts per nurse	18.01	17.65	18.52
Clinical time (hours)	116.17	109.93	106.83

We summarized the results of the pilot study in Table 6. The percentage of nurses' productivity was 77%, 75%, and 68% in the internal medicine, surgical, and ICU, respectively.

**Table 6:** Productivity measures.

Units	Working hours based on Timex records Mean (SD)	Clinical working hours (per month)	Productivity: Clinical working hours/Timex‐recorded working hours	Productivity Clinical hours/paid hours
		Mean (SD)	Mean	Mean
Intensive care unit (ICU)	155.52 (11.52)	106.83 (8.65)	0.68	0.49
Internal medicine unit	150.27 (19.86)	116.17 (15.45)	0.77	0.53
Surgical unit	144.66 (16.29)	109.93 (13.06)	0.75	0.52

## 4 Discussion

Nurses' productivity is a critical concern for hospital managers, particularly in the context of persistent nursing staff shortages that cannot be easily addressed in the short term. To maintain service delivery under these constraints, managers often seek to improve efficiency by optimizing the use of available human resources. Enhancing the productivity of human resources for health (HRH) therefore represents a key strategy for addressing these challenges. Despite its importance, HRH productivity is not routinely measured or systematically assessed in most hospitals, limiting managers' ability to make evidence‐based decisions. Our study provides a structured approach to quantify nursing productivity using time‐based measures, offering preliminary insights into workload distribution and potential efficiency gains within hospital units.

An effective nursing productivity tool will lead to optimal outcomes and match the number and competency of nurses with patients' acuity. The literature introduced several measures like RVU, the number of encounters, the number of procedures, generated revenue, and total compensation to calculate productivity. The point is that many of the mentioned models do not represent the multidimensionality of nursing practice [27]. Also, several studies emphasized using patient acuity and allocation of resources to measure nursing productivity [28, 29].

Different healthcare systems employ various billing models that incorporate nursing activities and consistently account for nursing costs [30, 31, 32]. The design and implementation of these models are highly dependent on national health policies and the structure of the local payment system. While such models have the potential to improve reimbursement efficiency, the assessment of nursing and human resources for health (HRH) productivity is largely shaped by the payment mechanisms in each system. Consequently, productivity measures often reflect the incentives and constraints inherent in the prevailing reimbursement framework, rather than providing a uniform or comparable evaluation across settings.

In this study, we used two measures to calculate nursing productivity, based on their real activities during working shifts. According to the results of the current study, the mean productivity scores of the nurses were 77%, 75%, and 68% in the internal medicine, surgical, and ICU, respectively. Pickard defined RVUs as numerical values of provider work and expenses [33]. Kapu et al. [34] and Lopatina et al. [35] defined some measures like admissions and discharges number and length of hospitalization. We could not calculate the measures due to a lack of coding and a billing system in the health system. Then we undertook a time and motion study to extract how nurses spend their time on different activities. Although the nursing workload may fluctuate from patient to patient, shift to shift, and even unit to unit [36]. Nursing activities in the same unit for most patients are repetitive in terms of type and time. The nursing workload is a direct reflection of nurses' productivity that affects the delivery of patient care, patient safety, as well as patient satisfaction.

Based on our results, nurses spent 60% of their working time on direct patient care. Reducing spending time on non‐nursing activities is necessary to provide high‐quality care. This can lead not only to increase patients' satisfaction but also to improved nursing productivity.

This study focused on time‐based indicators to measure nursing productivity, and it did not incorporate qualitative measures of work value or perceived contribution. Future research should extend this pilot by integrating qualitative approaches to capture nurses' perspectives on “productive” versus “non‐productive” time. In addition, mixed‐methods studies linking observed time‐based metrics with patient outcomes—such as patient safety, quality of care, and patient satisfaction—would provide a more comprehensive understanding of productivity. By identifying and testing these indicators in real clinical settings, this study lays the groundwork for a broader program of research that bridges quantitative measurement and qualitative insight.

An important methodological consideration in time–motion studies is the potential influence of the Hawthorne effect, that is, the risk that participants alter their behavior because they are aware of being observed. In our study, nurses were directly observed for extended periods, which may have temporarily increased task efficiency or altered time allocation. However, participants may habituate to the observer's presence over time, which can reduce the impact of the Hawthorne effect. We attempted to mitigate this influence by distributing observations across multiple units and shifts, though it cannot be completely eliminated. Kurtz et. al., reported that differences in compliance across observation time—comparing the first 2 h with the last 6 h of shifts—were small and inconsistent, indicating that nurses' behavior did not significantly change due to being observed [37]. Some studies have suggested the use of video‐camera monitoring, monitoring and feedback using employee badges with radiofrequency, and other wearable technology as additional strategies to reduce observer effects [38, 39, 40]. Consequently, the productivity estimates reported in this study should be interpreted as reflecting observed performance under study conditions, which may differ slightly from routine, unobserved practice. Future studies could incorporate longer observation periods and triangulation with electronic activity logs or automated tracking systems to further minimize observation‐related behavior changes and provide a more accurate assessment of true productivity.

The findings demonstrate noticeable discrepancies between standard working hours, actual recorded working hours (based on Timex records), and paid hours across all units. Working hours derived from Timex records represent the actual time nurses were physically present and working. However, these recorded hours were consistently lower than the standard working hours. This difference may partly reflect institutional policies that allow reduced required working hours for nurses with longer work experience or specific employment conditions. In contrast, paid hours were higher than both standard and recorded working hours in all units. This discrepancy is primarily attributable to payroll regulations. For example, night shifts are 12 h in duration but are compensated as 19 h, resulting in paid hours exceeding actual working time.

The majority of the reviewed studies employed quantitative survey designs and relied on basic data collection methods, such as questionnaires, self‐reported measures, or routinely recorded administrative data. In contrast, the present study emphasizes direct observation to accurately capture nursing activities as they occur, reducing bias inherent in self‐reports and administrative records. Data were collected across different clinical units, including surgical, internal medicine, and intensive care, to ensure a comprehensive assessment of nursing productivity in diverse practice settings.

### 4.1 Implications for Research and Practice

Familiarity with prior studies highlighted several limitations in our work. First, it was not possible to blind the data collection process, which may have introduced observation‐related behavior changes. Second, the productivity measures were extracted solely from the literature, which has inherent limitations and does not capture all contextual factors influencing nursing performance. Third, due to the design and scope of the study, we were unable to incorporate quality‐of‐care metrics, risk adjustment, or patient acuity measures, which are essential for a comprehensive assessment of healthcare productivity. Also, in this study, inter‐rater reliability was evaluated using percentage agreement, which may overestimate true agreement in time‐based observational data. Finally, as this study was conducted in a single hospital, the generalizability of the findings is limited.

## 5 Conclusions

The average nurses' productivity in the internal unit was higher than in other units. The productivity index may serve as a reference for performance‐based payment decisions. However, it is important to note that our assessment was based solely on time utilization and did not incorporate quality‐of‐care indicators or patient outcomes. Therefore, while efficiency appears favorable, the results should be interpreted with caution. Future studies are needed to integrate quality metrics and outcome measures alongside time‐based productivity indicators to provide a more comprehensive evaluation of nursing performance.

This research was financially supported by Ahvaz Jundishapur University of Medical Sciences. The funder had no role in the design of the study, data collection, analysis or interpretation of data, decision to publish or preparation of the manuscript (Contract Number: U‐99369).

## Author Contributions

**Elahe Farzan:** data curation, validation, formal analysis. **Zhila Najafpour:** methodology, conceptualization, investigation, visualization, writing – original draft, supervision, formal analysis. **Efat Jahanbani:** supervision, writing – review and editing. **Mohamad Arezomand:** data curation, writing – review and editing.

## Ethics Statement

Written informed consent was obtained from each participant before starting the study. Ethics approval was obtained from the Ethical Review Committee of Ahvaz Jundishapur University of Medical Sciences. This study was conducted under the principles of the Helsinki declaration (ethics code: IR. AJUMS.REC.1399.91).

## Conflicts of Interest

The authors declare no conflicts of interest.

## Transparency Statement

Dr. Zhila Najafpour affirms that this manuscript is an honest, accurate, and transparent account of the study being reported, that no important aspects of the study have been omitted; and that any discrepancies from the study as planned have been explained.

## Supporting information


Supporting File

## Data Availability

All data analyzed during this study are included in this published article and its supplementary information files. Any further information is available from the corresponding author on reasonable request.
