# Supplementary material for: Measuring Nursing Productivity Based on a Review of the Literature: A Pilot Study
Source: Health Sci Rep. 2026 Sep 9;9(9):e73218. doi: 10.1002/hsr2.73218 (PMC13559017; doi:10.1002/hsr2.73218)
Supplement: Supplementary file 1 [file HSR2-9-e73218-s001.docx]

**Supplementary 1: search strategy**

| Supplementary table 1: PubMed search ( 2021.06.02) | | |
| --- | --- | --- |
| 1. | **health Personnel[MeSH Terms]** | 530272 |
| 2. | **Health Manpower[MeSH Terms]** | 13422 |
| 3. | **health professional[MeSH Terms]** | 530273 |
| 4. | **health workforce[MeSH Terms]** | 13422 |
| 5. | **human resource[MeSH Terms]** | 76827 |
| 6. | **((health Personnel[MeSH Terms] OR Health Manpower[MeSH Terms] OR health professional[MeSH Terms]) OR (nurs*[Title] OR midwife*[Title] OR midwives*[Title]) OR (health workforce[MeSH Terms] OR human resource[MeSH Terms]))** | 839300 |
| 7. | **(Productivity[Title/Abstract] OR Effectiveness[Title/Abstract] OR Efficacy[Title/Abstract] OR Efficient[Title/Abstract] OR efficiency [Title/Abstract])** | 2124619 |
| 8. | **(Indicator[Title] OR Index[Title] OR Track[Title] OR Monitor[Title] OR track*[Title] OR observe*[Title] OR metric*[Title] OR network[Title] OR monitor*[Title] OR evaluate*[Title] OR assess*[Title] OR measure*[Title] OR managing[Title] OR oversee*[Title] OR model*[Title] OR framework*[Title])** | 1704902 |
| 9. | **#6 AND #7 OR #8** | 4398 |

| Supplementary table 2: SCUPUS (2021.06.02) | |
| --- | --- |
| TITLE-ABS-KEY ( ( manpower  OR  "health care manpower"  OR  "healthcare manpower"  OR  "medical manpower"  OR  "health manpower" )  OR  ( staff  OR  "health care staff"  OR  "healthcare staff"  OR  "medical staff"  OR  "health staff" )  OR  ( personnel*  OR  "health care personnel"  OR  "healthcare personnel"  OR  "medical personnel"  OR  "health personnel" )  OR  ( employe*  OR  "health care employe*"  OR  "health employe*"  OR  "healthcare employe*"  OR  "medical employe*" )  OR  ( worker*  OR  "healthworker*"  OR  "health care worker*"  OR  "healthcare worker*"  OR  "medical worker*" )  OR  ( workforce*  OR  "health care workforce*"  OR  "healthcare workforce"  OR  "health workforce"  OR  "medical workforce" )  OR  ( "health professional*"  OR  "health care professional*"  OR  "healthcare professional*"  OR  "medical professional*" )  W/5  ( productivity  OR  effectiveness  OR  efficacy  OR  efficient  OR  efficiency ) )  AND TITLE-ABS-KEY ( nurs*  OR  doctor*  OR  physician*  OR  midwife*  OR  midwives* )  OR  TITLE-ABS-KEY ( ( productivity  OR  effectiveness  OR  efficacy  OR  efficient  OR  efficiency )  W/5  ( nurs*  OR  doctor*  OR  physician*  OR  midwife*  OR  midwives* ) )  AND  TITLE-ABS-KEY ( ( indicator  OR  index  OR  track  OR  monitor  OR  track*  OR  observe*  OR  metric*  OR  network  OR  monitor*  OR  evaluate*  OR  assess*  OR  measure*  OR  managing  OR  oversee* OR model* OR framework*)  W/5  ( nurs*  OR  doctor*  OR  physician*  OR  midwife*  OR  midwives* ) ) | 478 |

| Supplementary table 3: Embase ( 2021.06.02) | | |
| --- | --- | --- |
| 1. | (staff/de OR 'health care personnel'/de OR 'nursing staff'/de OR manpower/de OR 'health care manpower'/de) OR (((personnel* OR employe* OR worker* OR ‘workforce*’ OR manpower OR staff* OR professional* OR hr) NEAR/3 (healthcare OR Health* OR care* OR medical* OR nurs* OR doctor* OR physician* OR midwife* OR midwives*)) OR (Human NEAR/3 resourc*)):ti,ab,kw | 2003519 |
| 2. | (‘nurs*’ OR ‘doctor*’ OR ‘physician*’ OR ‘midwife*’ OR ‘midwives*’): ti | 450109 |
| 3. | #1 or #2 | 2217572 |
| 4. | ((indicator OR index OR track OR monitor OR track* OR observe* OR metric* OR network OR monitor* OR evaluate* OR assess* OR measure* OR managing OR oversee* **OR mode OR framework**) NEAR/3 (nurs* OR doctor* OR physician* OR midwife* OR midwives*)):ti,ab,kw | 76975 |
| 5. | ((productivity OR effectiveness OR efficacy OR efficient OR efficiency) NEAR/3 (nurs* OR doctor* OR physician* OR midwife* OR midwives*)):ti,ab,kw | 6811 |
| 6. | #3 AND #4 AND #5 | 1070 |

| Supplementary table 4: ProQuest ( 2021.06.02) | | |
| --- | --- | --- |
|  | [ab(((manpower OR "health care manpower" OR "healthcare manpower" OR "medical manpower" OR "health manpower") OR (staff OR "health care staff" OR "healthcare staff" OR "medical staff" OR "health staff") OR (personnel* OR "health care personnel" OR "healthcare personnel" OR "medical personnel" OR "health personnel") OR (employe* OR "health care employe*" OR "health employe*" OR "healthcare employe*" OR "medical employe*") OR (worker* OR "healthworker*" OR "health care worker*" OR "healthcare worker*" OR "medical worker*") OR (workforce* OR "health care workforce*" OR "healthcare workforce" OR "health workforce" OR "medical workforce") OR ("health professional*" OR "health care professional*" OR "healthcare professional*" OR "medical professional*") OR (nurs* OR doctor* OR physician* OR midwife* OR midwives*))) AND ab((productivity OR effectiveness OR efficacy OR efficient OR efficiency)) AND ab(((indicator OR index OR track OR monitor OR track* OR observe* OR metric* OR network OR monitor* OR evaluate* OR assess* OR measure* OR managing OR oversee* OR model* OR framework*) NEAR/3 (nurs* OR doctor* OR physician* OR midwife* OR midwives*)))](https://search.proquest.com/myresearch/savedsearches.checkdbssearchlink:rerunsearch/1888944/SavedSearches?t:ac=SavedSearches) * ) ) | 1021 |

**Supplementary 2:** Characteristics of included studies in the review phase

| **Supplementary Table 5: Studies included in the review phase (measurement of nurses' productivity)** | | | | | | |
| --- | --- | --- | --- | --- | --- | --- |
| **Row** | **Author** | **Type of study** | **Study title** | **Purpose of study** | **Publication year** | **Study place** |
| 1. | Holcomb | Qualitative | Defining and measuring nursing productivity: a concept analysis and pilot study | First: to report a concept analysis of nursing productivity show the complexity of the concept and its measurement. Then show how the concept analysis was used to design a method for measuring nursing productivity.  Third, to describe how used this measure in a pilot study of the impact of a differentiated Registered Nurse (RN) practice model on productivity.  Finally, identified the challenges encountered and present recommendations for nurse executives and researchers based on findings. | 2002 | USA |
| 2. | Forcheh | Quantitative | Determinants of effective productivity among service workers: the case of nurses in Botswana | Measure of effective productivity among service workers and seeks a parsimonious predictive model of “effective productivity” among nurses | 2007 | Botswana |
| 3. | Hinkel | Quantitative | Practice and Productivity of Physician Assistants and Nurse Practitioners in Outpatient Oncology Clinics at National Comprehensive Cancer Network Institutions | To define how National Cancer Institute (NCI) designated comprehensive cancer centers use Pas/NPs and to pilot a self-reported PA/NP productivity tool | 2010 | USA |
| 4. | Moote | Quantitative | Productivity Assessment of Physician Assistants and Nurse Practitioners in Oncology in an Academic Medical Center | To quantify the productivity of Pas and NPs working in oncology in an academic medical center | 2012 | USA |
| 5. | Ragab | Quantitative | Nurses’ Workload and its’ Impact on Productivity in Trauma and Post-Operative Intensive Care Units at Assiut University Hospital | Measuring nurses’ workload at trauma and post-operative intensive care units and identifying the impact of nurses’ workload on nursing productivity | 2013 | Egypt |
| 6. | Souza | Quantitative | Measurement of nurses’ workload in an oncology outpatient clinic | Measuring the workload and productivity of nurses in an oncological outpatient service | 2013 | Brazil |
| 7. | Rashed. S | Quantitative | Measuring nurses’ productivity at Assiut University Hospital | Measuring nurses’ productivity | 2015 | Egypt |
| 8. | McDonnell | Quantitative | Relative Productivity of Nurse Practitioner and Resident Physician Care Models in the Pediatric Emergency Department | To determine the relative clinical productivity of PED attending physicians working with residents compared with PED attending physicians working with nurse practitioners (NPs). | 2015 | USA |
| 9. | Moran | Quantitative | PA and NP productivity in the Veterans Health Administration | Evaluation of factors affecting the productivity of physician assistants (Pas) and nurses (NPs) | 2016 | USA |
| 10. | Hunt | Descriptive (Text/opinion) | Meeting the measurements of inpatient staffing productivity | Discusses how productivity is calculated, also gives ideas on how to keep team in the green of productivity. | 2018 | USA |
| 11. | Lucatorto | Descriptive | Nurse practitioner productivity measurement: An organizational focus and lessons learned | Providing a previous review of productivity measurement, how to measure productivity in the Veterans Health Administration and providing guidance on how to measure and use the productivity of clinical nurses. | 2019 | USA |

**Supplementary 3: Risk of bias assessment for included studies** **based on JBI scale**

| Table 6: Risk of bias assessment for included studies based on JBI scale | | | | | | | | | | |
| --- | --- | --- | --- | --- | --- | --- | --- | --- | --- | --- |
| Row | Author | Appraisal tool | Q1 | Q2 | Q3 | Q4 | Q5 | Q6 | Q7 | Q8 |
| 1 | Souza | Quantitative | Yes | Yes | Yes | Yes | No | No | Yes | Yes |
| 2 | Hinkel | Quantitative | Yes | Yes | Yes | Yes | No | No | Yes | Yes |
| 3 | Holcomb | Quantitative | Yes | Yes | Yes | Yes | Yes | Yes | yes | Yes |
| 4 | Ragab | Quantitative | Yes | Yes | Yes | Yes | No | No | Yes | No |
| 5 | Rashed | Quantitative | Yes | Yes | Yes | Yes | No | No | Yes | No |
| 6 | Moote | Quantitative | Yes | Yes | Yes | Yes | Yes | Yes | Yes | Yes |
| 7 | McDonnell | Quantitative | Yes | Yes | Yes | Yes | Yes | Yes | Yes | Yes |
| 8 | Forcheh | Quantitative | No | No | Yes | Yes | Yes | Yes | Yes | Yes |
| 9 | Moran | Quantitative | Yes | No | Yes | Yes | Yes | Yes | Yes | Yes |
| 10 | Lucatorto | Text and Opinion / Expert Opinion | Yes | Yes | Yes | Yes | Yes | Unclear | - | - |
| 11 | Hunt | Text and Opinion / Expert Opinion | Yes | Yes | Yes | Yes | Yes | Unclear | - | - |

**Supplementary 4:** Nurses’ activities

Nurses’ activities were grouped into four categories including a direct patient care, collective patient care, unit-related, personal-related, and other tasks.

- Direct patient care is defined as care that can be directly related to one specific patient and involves: Communication between the nurse and the patient. This category includes 14 activity groups (i.e., handing out medication, wound care, communication with patients or their families, and so on). At the same time, indirect nursing care is the specific patient-related services that do not require communication between the nurse and patient. This category includes documentation and communication with other units).
- Collective patient care is defined as patient-related tasks that are impossible to attribute to a specific patient. This category includes three activity groups (i.e., general preparation of medication, specimen collection, and patient handover).
- Unit-related tasks include five activity groups (i.e., education, meetings, administrative duties, domestic duties, checking mobile trolley, and incident reporting).
- Personal-related tasks include three activity groups (such as breaks, walking time, and others)

**Supplementary 4**

**Table 7: nurses’ activities**

**Patient direct care**

Ventilatory support, i.e., Respiratory support, care of artificial airways, improving lung function

Monitoring the provision of the patient's excretory needs*(catheter, bladder washing, drain care; lavage and ostomy care, chest tube)

infusion of intravenous fluids, serum therapy, blood transfusion, and enteral feeding

Assisting with procedures/investigations, e.g., line insertion, intubation, CXR

Skin care

Collecting, labeling and dispatch of laboratory samples

Gastric lavage and gavage

Drug administration

Cognitive stimulation

Observing vital signs and input-output

Transferring a patient to other hospitals, wards, scan or operating room

Support and care for the family and patient

Wound care and dressings

Mobilization and positioning

Performing hygiene procedures

Assisting with procedures/investigations, e.g., line insertion, intubation, CXR

Preparation of patient before and after clinical tests and medical/surgical procedures and follow up patient consultations

patient initial assessment and re-assessment

Developing nursing care plan

Reporting, documentation, ordering, communications

- Requesting tests, imaging, blood reservation
- Follow-up the patient's paraclinical procedures (scopies, imaging)
- Follow up patient consultations
- Nursing report documentation
- Admission and discharge documentation
- Medicating documentation
- Recording of vital signs and Input output charting
- Checking Patient medical record

**Collective patient care**

checking trolley code

Patient handover

Drug preparation

Sample preparation

**Unit-centered**

Meetings, training, , incidence reporting and patient safety related affaires, students training, communication with colleges, answering the phone

**Personnel-centered**

Eating, phoning, relaxing, changing dress, …
